# Multidrug resistance transporter-1 dysfunction perturbs meiosis and Ca^2+^ homeostasis in oocytes

**DOI:** 10.1530/REP-22-0192

**Published:** 2022-10-10

**Authors:** Dalileh Nabi, Davide Bosi, Neha Gupta, Nidhi Thaker, Rafael Fissore, Lynae M Brayboy

**Affiliations:** 1 Department of Neuropediatrics Charité-Universitätsmedizin Berlin, corporate member of Freie Universität Berlin, Humboldt-Universität zu Berlin, and Berlin Institute of Health, Berlin, Germany; 2 Klinik für Pädiatrie m. S. Neurologie, Charité Campus Virchow Klinikum, Berlin, Germany; 3 Department of Veterinary and Animal Sciences, University of Massachusetts, Amherst, Massachusetts, USA; 4 Department of Reproductive Biology, Bedford Research Foundation, Bedford, Massachusetts, USA

## Abstract

**In brief:**

Oocyte quality remains the most important and unsolved issue in reproduction. Our data show that multidrug resistance transporters and oocyte mitochondria are involved in determining oocyte quality in a mouse model.

**Abstract:**

Multidrug resistance transporter-1 (MDR-1) is a transmembrane ATP-dependent effluxer present in organs that transport a variety of xenobiotics and by-products. Previous findings by our group demonstrated that this transporter is also present in the oocyte mitochondrial membrane and that its mutation led to abnormal mitochondrial homeostasis. Considering the importance of these organelles in the female gamete, we assessed the impact of MDR-1 dysfunction on mouse oocyte quality, with a particular focus on the meiotic spindle organization, aneuploidies, Ca^2+^ homeostasis, ATP production and mtDNA mutations. Our results demonstrate that young *Mdr1a* mutant mice produce oocytes characterized by lower quality, with a significant delay in the germinal vesicle to germinal vesicle breakdown transition, an increased percentage of symmetric divisions, chromosome misalignments and a severely altered meiotic spindle shape compared to the wild types. Mutant oocytes exhibit 7000 more SNPs in the exomic DNA and twice the amount of mitochondrial DNA (mtDNA) SNPs compared to the wild-type ones. Ca^2+^ analysis revealed the inability of MDR-1 mutant oocytes to manage Ca^2+^ storage content and oscillations in response to several stimuli, and ATP quantification shows that mutant oocytes trend toward lower ATP levels compared to wild types. Finally, 1-year-old mutant ovaries express a lower amount of SIRT1, SIRT3, SIRT5, SIRT6 and SIRT7 compared to wild-type levels. These results together emphasize the importance of MDR-1 in mitochondrial physiology and highlight the influence of MDR-1 on oocyte quality and ovarian aging.

## Introduction

The reproductive life span of a woman lasts for a definite period of time, from the age of menarche at approximately 12 years of age until menopause at 49.9 years (Appiah *et al.* 2021) due to age-related decline of oocyte quality. During folliculogenesis, the oocyte undergoes exponential growth during which it acquires the competency to resume meiosis, evidenced by germinal vesicle breakdown (GVBD) (Pincus & Enzmann 1935, Edwards 1965). In a fully grown follicle and following the luteinizing hormone surge, oocytes resume meiosis, undergo GVBD and extrude the first polar body (PBI). At this point, oocytes arrest at metaphase II (MII) awaiting fertilization. After fertilization, the oocyte will complete the second meiotic division and extrude the second polar body (PBII) and commence embryo development (Talbot *et al.* 2003). However, with poor oocyte quality – a central hallmark of diminished ovarian reserve (DOR) or the stock of immature oocytes and their adjacent somatic cells collectively known as primordial follicles – these processes are compromised. Ovarian reserve determines the fertile life span of a woman (Wallace & Kelsey 2010, Findlay *et al.* 2015). Clinically, DOR is an ill-defined, common diagnosis consisting of decreased quantity and quality of oocytes characterized by a decrease in anti-Müllerian hormone levels, an increase in follicle-stimulating hormone levels and a decrease in antral follicular count (Sills *et al.* 2009). The American Society for Reproductive Medicine states, ‘there is no uniformly accepted definition’ (Hansen 2008). It should be noted that reproductive aging does not always occur chronologically. While DOR presents a significant obstacle for women of advanced maternal age, which is greater than 35 years old, younger women suffer from DOR too (Devine *et al.* 2015). Approximately 10% of patients presenting to infertility specialists have DOR (Scott & Hofmann 1995), and DOR has increased 42% among women under 40 (Devine *et al.* 2015).

Oocyte quality is truly the ability of the egg to be fertilized, mature to MII and give rise to healthy offspring (Coticchio *et al.* 2004). These processes are energy driven by oocyte mitochondrial efficiency (Kirillova *et al.* 2021). The oocyte has more mitochondrial abundance compared to somatic cells (Q. Wang *et al.* 2019). Our work has shown previously that oocyte mitochondria express multidrug resistance transporter-1 (MDR-1) (Clark *et al.* 2019), an ATP-dependent transporter involved in the efflux of xenobiotics (Linton 2007, Wolf *et al.* 2012) and toxicants out of the oocyte (Brayboy *et al.* 2013, p. 20). We have also shown that MDR-1 is present in the oocyte mitochondrial membrane, and its dysfunction leads to aberrant oocyte mitochondrial physiology and metabolism (Clark *et al.* 2019). Here, we have studied the effects of MDR-1 dysfunction on the overall oocyte quality. This manuscript demonstrates that dysfunctional MDR-1 is associated with meiotic defects. Specifically, energy-consuming processes which are mitochondrial dependent such as meiotic resumption, spindle formation and chromosome alignment are negatively impacted. Not only does MDR-1 dysfunction impact oocyte quality, but it also appears to accelerate the onset of ovarian aging, as evidenced by altered sirtuins (SIRTs), important regulators of metabolism and redox state of the cell (Nemoto *et al.* 2005, Sosnowska *et al.* 2017, Singh *et al.* 2018). Our overall goal in this manuscript was to evaluate the association of MDR-1 mutation with markers of oocyte quality and ovarian aging.

## Materials and methods

### *Mdr1* mutant

Wild-type (CF-1) and P-glycoprotein (from this point on referred as *Mdr1*) mutant CF-1 mice were originally obtained from Charles River Laboratories and were inbred for multiple generations. Littermate controls were used in all experiments. In our study, we used 8- to 20-week-old wild-type and mutant CF-1 females except for aging studies in which 9- to 12-month-old mice were used. All mice were bred and maintained under a 12 h light/12 h darkness cycle at 22°C (± 2 °C) and 55% humidity (±5%) in the animal facility of Charité–Universitätsmedizin Berlin, according to approved institutional guidelines. All animal experiments were performed according to the relevant ethical requirements and were approved by the state animal welfare office (license numbers H 20/21 and TCH 20/21).

### Genotyping

Mouse genotyping was performed by digesting mouse ear samples with DirectPCR Lysis Reagent (Viagen, Cedar Park, Tx, USA; Cat. #102-T) containing 0.5–1 mg/mL Proteinase K (BioLabs, Cat. #P8107S) overnight at 55°C. Lysates were then incubated at 85°C for 45 min. PCRs were performed on a Biometra-Tone 96G (Analytikjena, Göttingen, Lower Saxony, Germany). Three-primer touchdown PCR was used to amplify the region between exon 22+23 of wild-type genomic DNA and murine leukemia virus insertion (MuLV)+exon 23 in the case of *Mdr1a* mutant DNA. Primers used were: forward 1 5’-CTT TGA CTC GGG AGC ATT C-3’, forward 2 5’-ATC AGC GAG ACC ACG ATT C-3’ and reverse 5’-TGA GTT GTT GTG TCA CCA AGT A-3’.

### Isolation, culture and live imaging of mouse oocytes

Ovaries were isolated in M2 medium (Sigma, Cas. #M7167-100ML) supplemented with 2.5 μM milrinone (Sigma, Cas. #78415-72-2) and punctured with a 27-gauge needle to release the oocytes. All experiments were conducted by the same two people with years of experience in murine oocyte isolation. Wild-type and *Mdr1* mutant COCs that were retrieved were denuded using gentle mouth pipetting. Very small oocytes already denuded were excluded from experimentation. For each experiment, the same number of ovaries was used for each genotype. GV stage oocytes were maintained at prophase arrest in the M2 medium with milrinone under paraffin oil (Sigma, Cas. #8012-95-1) at 37°C. For analysis of GVBD and polar body extrusion (PBE), oocytes were released into the M2 medium, and the oocytes from each genotype were then taken to the imaging facility and located under the microscope for approximately 30 min (time 0) and imaged every 30 min for up to 20 h on an inverted Leica Thunder Imager DMi8 microscope with a Plan APO 20×/0.75 NA objective, an sCMOS DFC9000 GT Microscope Camera (Leica), standard filter sets and an environmental chamber to maintain 37°C. Z-series optical stacks of 80 µm were recorded with slices taken every 7 μm. All images were acquired using the Leica Application Suite X (LAS X) software and analyzed with Fiji imageJ.

To obtain MII oocytes, we performed superovulation. Specifically, wild-type and *Mdr1* mutant CF-1 mice were injected with 5 IU pregnant mare serum gonadotropin 48 h prior to 5 IU human chorionic gonadotropin (hCG) injection. Fourteen hours post-hCG mice were sacrificed with cervical dislocation. Then, a thoracotomy was performed, and the abdominal cavity of the animals was then opened with a vertical incision. The horn and oviducts were identified, and the ampullae were opened with a linear salpingostomy technique using a 27-gauge needle releasing the MII oocytes in the M2 medium. Cumulus cells were quickly removed with M2 + hyaluronidase medium (CytoSpring, Mountain View, CA, USA; Cat. #M2102HV). The denuded MII oocytes were then quickly transferred into the M2 medium for counting.

### Oocyte chromosome spreads

Oocyte chromosome spreads were prepared according to an adapted method from Tarkowski (1966). *In vitro* matured oocytes were treated with Tyrode’s solution (Sigma, Prod. #T1788) to remove the zona pellucida. Oocytes were then washed and incubated in 1% sodium citrate solution for 5–10 min. A microdrop of sodium citrate was formed in the middle of a microscope slide, and a group of oocytes were placed in it. One drop of Carnoy’s solution (ethanol:acetic acid (3:1)) was dropped on the oocytes. After the oocytes settled down, another drop of Carnoy’s solution was added followed bythree to four more drops. The slides were dried and prepared for immunostaining.

### Immunofluorescence and image acquisition

For whole-mount immunofluorescence staining, oocytes were cultured in M2 plus or minus 2.5 μM milrinone and collected at the relevant time points. The zona pellucida was removed using the Tyrode’s solution. After a 30-min recovery period, the oocytes were fixed in 2% formaldehyde and 0.1% Triton X-100 in M2 media for 10 min. Cells were washed once in blocking buffer (3–5% BSA and 0.1% Triton X-100 in PBS), before incubation in blocking buffer for 30 min at room temperature (RT). The cells were transferred into the primary antibody solution (3% BSA and 0.1% Triton X-100) and incubated at 4°C overnight. The primary antibody used was a rabbit anti-β-tubulin (Cat. #ab6046; Abcam) at 1:1000. The secondary antibody used was anti-rabbit Alexa Fluor 568 (A11036; Invitrogen) at 1:500. The cells were washed at RT in 0.1% Tween 20 in PBS 3 × 5 min and mounted in Vectashield (Vector Laboratories; H-1000) plus 1 μg/mL DAPI for imaging. Fixed oocytes were imaged with an inverted Leica Thunder Imager DMi8 microscope using a 63 x/1.4 NA oil objective and a sCMOS DFC9000 GT Microscope Camera (Leica) and by a Nikon A1Rsi+ Scanning Confocal Microscope (Nikon Europe B.V.) using a 63 x/1.27 NA oil objective. The brightness and contrast of the images were modified for image preparation.

#### Oocyte immunoblot

In order to localize MDR-1 expression in oocytes, for each sample, 75 GV oocytes were isolated and pooled from 3 unstimulated mice aged 4–5 months from both mutant and wild-type animals using the protocol described by Liu *et al.* (2018). Denuded GV oocytes were washed in three small drops of PBS, collected and released in a small Eppendorf tube containing 15 µL of SDS lysis buffer. To ensure oocyte lysis, three cycles of freeze–thawing were performed. Lysates were then stored at –80°C. Protein samples were prepared by adding 5 µL of 2× Sample Laemmli buffer (4% SDS, 20% glycerol, 0.004% bromphenol blue, 0.125 M Tris–Cl pH 6.8, 10% 2-mercaptoethanol, (US Biological, Salem, MA, USA)) and centrifuged for 10 s at 6500 ***g***. Samples were loaded and separated onto a 12% SDS gel. Gels were run at 125 V for 120 min. PageRuler™ Prestained Protein Ladder (Cat. #26616m, Fisher Scientific) was used to identify protein kDa. Proteins were transferred to nitrocellulose for 70 min at 30 V, and blots were blocked for 1 h in 5% non-fat milk/TBS-T (w/v) at RT. Membranes were cut at 70 KDa. The upper parts were labeled overnight at 4°C with rabbit recombinant anti-P glycoprotein MAB (Abcam, Cat. #ab170904), with 1:500 dilution in TBS-T; the bottom parts were labeled overnight at 4°C with mouse anti-GAPDH mAb (Cat. #AM4300, Applied Biosystem), with 1:1000 dilution in 5% milk/TBS-T. Anti-rabbit IgG (H+L) poly-HRP (Cat. #32260, Invitrogen), with 1:10,000 dilution in TBS-T and anti-mouse IgG HRP conjugated (Cat. #DC02L, Calbiochem), with 1:2000 dilution were used as secondary antibodies. After three washes, membranes were incubated with secondary antibody solution for 1 h at RT. Bands were visualized by chemiluminescence with Amersham ECL SelectTM Western blotting detection reagent (Cat. #RPN2235, Cytiva, Freiburg, Breisgau, Germany).

#### Ovarian immunoblot

Ovaries from 9- to 12-month old animals were collected and homogenized in 500 µL lysis buffer (50 mM Tris pH 8.0, 150 mM NaCl, 0.5% sodium deoxycholate, 1% NP-40, 0.1% SDS, 5 mM NaOV4, 10 mM NaF, and protease inhibitors (11697498001, Roche)) by a pestle and kept for 30 min on ice. After centrifugation for 20 min at 18,400 ***g*** at 4°C, the supernatant was collected and used as total cell extract. Protein concentrations of the individual samples were calculated using the Bio-Rad protein assay (Bio-Rad, Cat. #5000002), a colorimetric assay for measuring the total protein concentration. The absorbances of several standard samples with known concentrations were calculated using Thermo Helios Alpha UV/Vis Spectrophotometer (Thermo Fisher Scientific), and a standard curve was obtained. Then, the absorbance of our unknown samples was obtained, and protein concentration was derived from the standard curve. Twenty micrograms of proteins were loaded and separated on a 12% SDS gel. PageRuler™ Prestained Protein Ladder (Cat. #26616m, Fisher Scientific) was used to identify protein kDa. The gels were then run at 125 V for 60 min. Gels were then transferred to a PVDF membrane activated with methanol using the Mini Trans-Blot Electrophoretic Transfer Cell (Bio-Rad) for 2 h at 100 V. Membranes were then blocked with 5% (w/v) milk/TBS-T for 1 h and then were incubated with the primary antibody overnight at 4°C. Primary antibodies used were as follows: rabbit anti-Sirt1 (Cat. #9475); rabbit anti-Sirt3 (Cat. #5490); rabbit anti-Sirt5 (Cat. #8779); rabbit anti-Sirt6 (Cat. #12486); rabbit anti-Sirt7 (Cat #5360) (Cell Signaling Technology) at 1:500 in TBS-T. Due to the similar molecular weights of GAPDH (our loading control) with most sirtuins, the membranes were cut into strips, stripped using of ROTI Stripping Puffer (#0083.1, Roth, Karlsruhe, Baden-Württemberg, Germany) in a water bath rocking at 56°C for 30 min and washed with TBS-T and imaged to ensure there were no residual sirtuin bands. After blocking the strips of membrane, they were incubated with mouse anti-GAPDH (Cat. #AM4300, Applied Biosytem) at 1:1000 dilution in 5% (w/v) milk/TBS-T. Secondary antibodies were used as follows: anti-rabbit IgG HRP conjugated (Cat. #32260; Invitrogen) at 1:10,000; anti-mouse IgG HRP conjugated (Cat. #DC02L, Calbiochem) diluted 1:2000. After three washes in TBS-T, secondary antibody incubation was performed for 1 h at RT. Following three washes, proteins were visualized by chemiluminescence with Amersham ECL Select^TM^ western blotting detection reagent (Cat. #RPN2235, Cytiva). Band intensity was calculated using ImageJ software, and the ratio of sirtuins/GAPDH expression was normalized.

### Calcium homeostasis in oocytes

Superovulated oocytes were collected and incubated in potassium simplex optimized media (KSOM) at 37°C in a humidified atmosphere of 5% CO_2_ for at least 1 h. Ca^2+^ imaging was performed as described earlier in Ardestani *et al.* (2020). Briefly, oocytes were incubated with Ca^2+^ sensitive dye Fura-2-acetoxymethyl ester (Fura-2AM, Invitrogen, F1221) for 20 min at RT. Stained oocytes were placed in Tyrode's Lactate (TL)-HEPES with or without Ca^2+^ on a glass-bottom dish (Mat-Tek Corporation, Ashland, MA, USA) covered with mineral oil. Oocytes were monitored using an inverted microscope (Nikon) for fluorescence measurements. Fura-2AM was excited between 340 and 380 nm wavelengths, and the fluorescence was captured every 20 s. The data obtained were analyzed using Microsoft Excel and GraphPad Prism version 5.0 (GraphPad Software). The assessment of Sr^2+^ influx and oscillations were carried out in Ca^2+^-free TL-HEPES supplemented with 10 mM strontium chloride hexahydrate (Cat No. 255521, Sigma-Aldrich) throughout the monitoring. Ca^2+^ store content was assessed by using Ca^2+^-free TL-HEPES followed by the addition of ionomycin (IO) (2.5 µM) or thapsigargin (TG) (10 µM) at the indicated time points. For *Plc𝜁1*-induced calcium oscillations, *Plc𝜁1* plasmid was linearized with Pme1 restriction enzyme, and the mRNA was synthesized using the mMESSAGE mMACHINE T7 kit (Invitrogen, AM1344) according to the manufacturer’s protocol. A Poly(A)-tail was added to the mRNA using Pol(A) Tailing Kit (Invitrogen, AM1350). About 0.01 µg/µL of *Plc𝜁1* mRNA was microinjected in the oocytes and Ca^2+^ was monitored in TL-HEPES with minimal Ca^2+^, as described earlier.

### β-Galactosidase staining

Ovaries were collected, quickly washed with PBS, embedded in OCT, frozen and stored at –80°C. Cryostat sections (7 μm) were mounted on Superfrost Plus slides (Fisher Scientific), fixed with 1% paraformaldehyde and stained by incubation overnight at 37°C in a 4 mg/ml X-Gal following the manufacturer’s protocol (AssayGenie, Cat. #CV0018). Sections were counterstained with natural red (Sigma) and dehydrated by repeated immersions in ethanol 70%, 96% and 100% for 2 min and a final immersion in xylene for 10 min. Ovarian sections were visualized under dissection microscope, and X-Gal positive follicles were counted.

### Measurement of oocyte ATP content

GV oocytes were isolated from the ovaries of 2- to 4-month old mice (*n*  = 6 wild type and *n*  = 6 *Mdr1* mutants). Ten oocytes per mouse (*n*  = 60 wild type and *n*  = 60 *Mdr1* mutants) from each genotype were quickly washed in Dulbecco’s phosphate buffered saline (DPBS) 1× (Gibco, Cat. #14190-094) drops. Oocytes were transferred into 1.5 mL Eppendorf tubes containing 100 µL of Releasing Reagent (Sigma-Aldrich, Cat. #FLSAR) and put on ice for about 5 min. The ATP quantification was performed quickly using ATP Bioluminescent Somatic Cell Assay Kit (Sigma-Aldrich, Cat. #FLASK), in a 96-well plate following the manufacturer’s instructions.

### Whole exome and mitochondrial DNA sequencing

MII oocytes were isolated from the oviducts after natural ovulation and snap-frozen in liquid nitrogen. Oocytes were then lysed, and the whole genome was amplified by using the ResolveDNA^TM^ Whole Genome Amplification kit (Prod. #100136, BioSkryb, Durham, NC, USA). Sequencing was performed by CeGat GmbH. The sequence read length was 2× 100 bp, and an output of 12 Gb (WES Classic) per sample was chosen. Demultiplexing of the sequencing reads was performed with Illumina bcl2fastq (2.20). Adapters were trimmed with Skewer (version 0.2.2) (Jiang *et al.* 2014). Quality trimming of the reads has not been performed. Trimmed raw reads were aligned to 10 mm using the Burrows-Wheeler Aligner (BWA-mem version 0.7.17-cegat). Variant calling was performed in the target regions with ± 30 base pairs. The lists also include variants with low frequencies (OFA down to 2% of sequenced reads). The quality of the FASTQ files was analyzed with FastQC (version 0.11.5-cegat).

### Statistical analysis

The data processing, statistical analysis and plotting were performed in Fiji, Excel (© 2015 Microsoft), and GraphPad Prism (GraphPad Software, Inc.). Error bars indicate means ± s.d.. Sample sizes and statistical tests are indicated in the figure legends. All experiments were performed with a minimum of three biological replicates from each genotype: three wild types and three *Mdr1* mutants.

## Results

### Mdr1 is expressed in wild-type and mutant GV oocytes

We localized the MDR-1 protein in the control and mutant groups. Our immunoblot experiment demonstrates the presence of MDR-1 in GV oocytes (Supplementary Fig. 4, see section on supplementary materials given at the end of this article).

#### Meiosis resumption and progression

**Figure 1:**
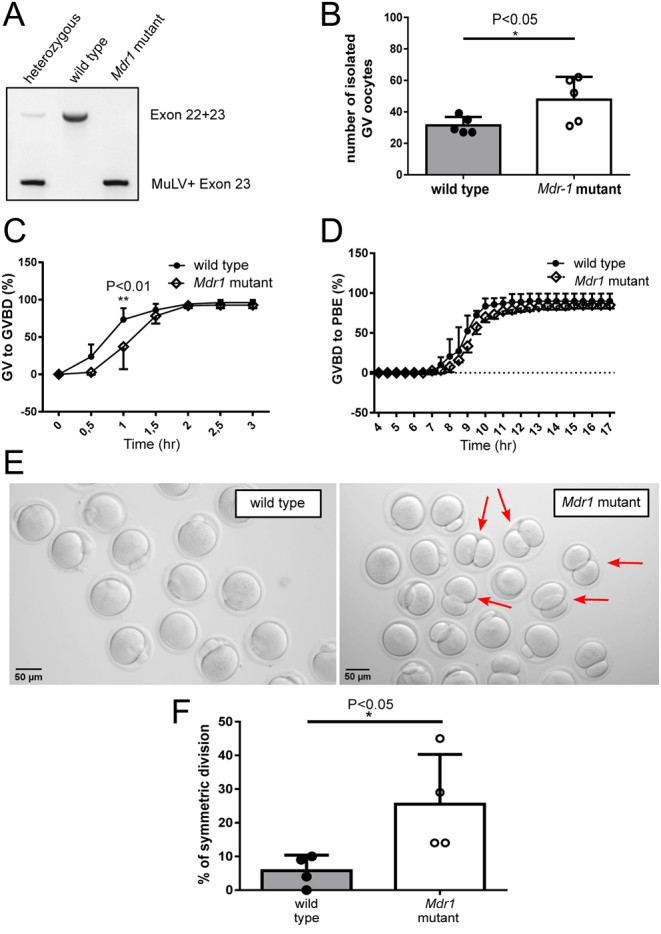
Two-month-old mutant oocytes show a delayed germinal vesicle breakdown (GVBD), and the resulting MII oocytes present enlarged first polar bodies (PBs). (A) Mouse genotyping. MuLV, murine leukemia virus insertion. (B) Number of GV oocytes isolated from wild-type and *Mdr1* mutant mice. Unpaired *t*-test; *P < 0.05. *n*  = 5 wild-type and 5 *Mdr1* mutant mice. (C) Percentage of young mutant and wild-type oocytes transitioning from germinal vesicle to germinal vesicle breakdown (GVBD) 30 min after experiment start and 60 min after milrinone removal. Both wild-type and mutant oocytes reach GVBD, although mutant oocytes are significantly delayed in the transition. Two-way ANOVA; **P < 0.01. (D) Percentage of oocytes extruding the first polar body (PBI) after 16 h of* in vitro* maturation. No difference was observed in the frequency and timing of polar body extrusion (PBE) between wild-type and mutant oocytes. *n*  =  3 wild-type and 3 *Mdr1* mutant mice. (E and F) Images representing the different PB sizes in wild-type and mutant oocytes. Oocytes with symmetric divisions are indicated by a red arrow. Unpaired *t*-test; *P < 0.05. Scale bar = 50 µm. *n*  = 3 wild-type and 3 *Mdr1* mutant mice.

To elucidate the relationship between *Mdr1a* mutation and oocyte phenotypes, we investigated the roles of MDR-1 during oocyte maturation. *Mdr1a* mutant gene carries an insertion in its exon 23 which causes a loss of function of the protein (Pippert & Umbenhauer 2001). Amplification of the insertion region by genomic PCR was used to distinguish between wild-type and mutant MDR-1 mice (Fig. 1A). A significantly higher number of GV oocytes were isolated from *Mdr1* mutant 2- to 3-month-old ovaries compared to the wild-type ones (*n*  = 31 ± 2.4 vs *n*  = 48 ± 7 in wild-type and mutant ovaries, respectively) (Fig. 1B). Live imaging of* in vitro* matured wild-type and *Mdr1* mutant oocytes showed that MDR-1 mutation did not affect meiotic resumption, as evidenced by the similar rates of GVBD after 3 h* in vitro* culture (Fig. 1C). However, we observed a significant delay in the mutant oocytes going through GVBD compared to the wild type (Fig. 1C, **P < 0.01), indicating a defect in the meiotic resumption. Subsequently, the rate of first PBE was evaluated in *Mdr1* mutant oocytes compared to the wild type (Fig. 1D). While we did not find a difference in the rate of PBE, we observed that oocytes with atypical symmetrical division were frequently detected in the mutant (Fig. 1E and F arrowhead, 5.8 ± 2.3% vs25.5 ± 7.4%, respectively, *P < 0.05) (Supplementary Video 1). These results indicate that *Mdr1* mutant oocytes have a deficiency in the first steps of meiotic resumption and its correct maturational progression.

#### MDR-1 deficiency leads to misaligned meiotic spindles in young oocytes

**Figure 2:**
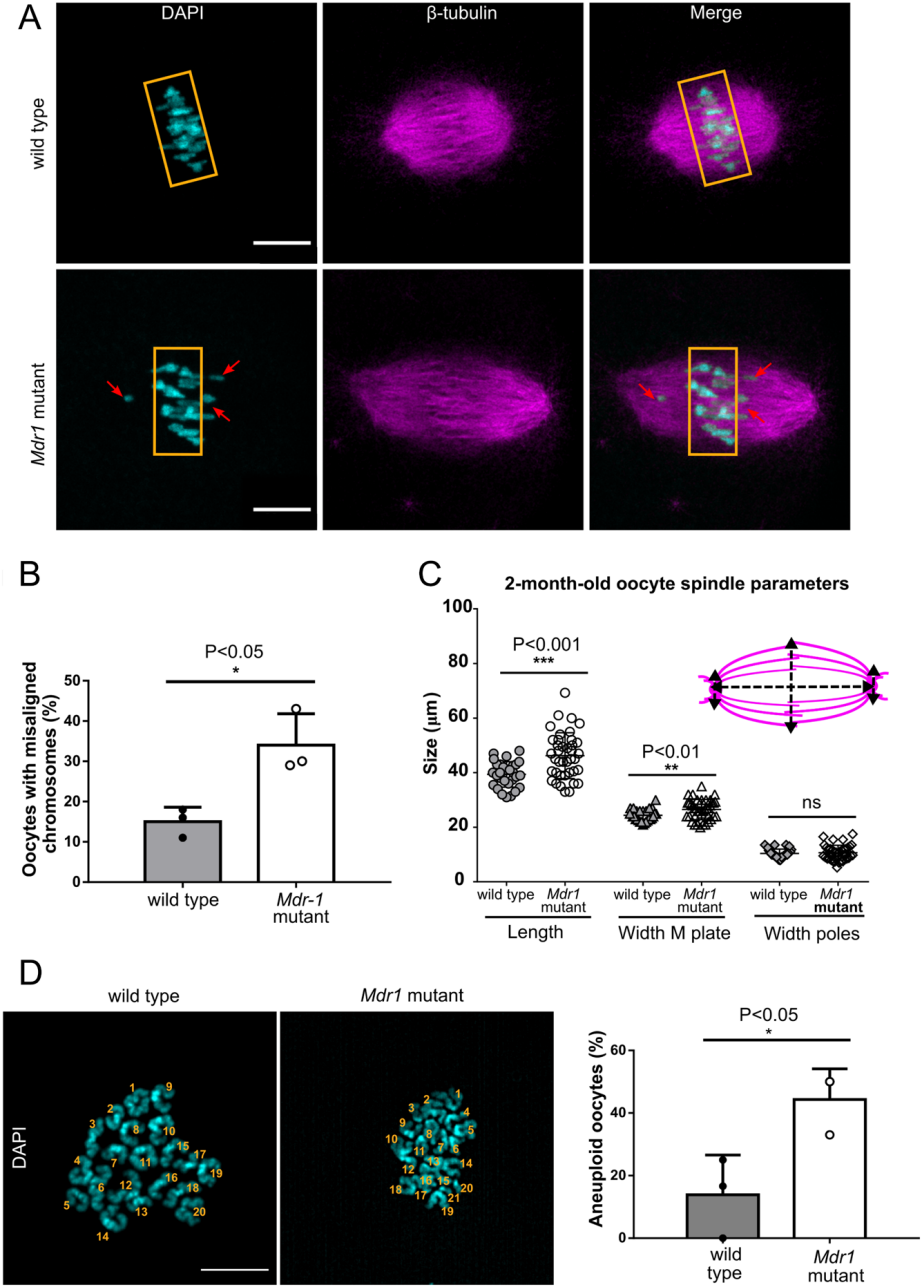
Two-month-old mutant oocytes present misaligned chromosomes and abnormal spindle shape. (A) Two-month-old *Mdr1* mutant oocytes have altered metaphase I (MI) chromosome alignment. Indeed, 34% of MI oocytes from 2-month-old mice present misaligned chromosomes, while only 15% of wild-type oocytes from age-matched controls have an aberrant chromosome alignment. *n*  =  3 wild-type and 3 *Mdr1* mutant mice. Whole-mounted oocytes were stained with anti-tubulin antibody (magenta) for the spindle and chromosomes were counterstained with DAPI (cyan). Red arrows indicate misaligned chromosomes. Scale bar = 20 µm. (B) Percentage of oocytes characterized by the presence of misaligned chromosomes in the transition from MI to MII stage. *n*  =  3 wild-type and 3 *Mdr1* mutant mice. Unpaired *t*-test; *P < 0.05. (C) Analysis of 2-month-old oocytes of spindle shape revealed that mutant oocytes present a significant alteration of spindle length (unpaired *t*-test, ***P < 0.001) and width (unpaired *t*-test, **P < 0.01) at M plate compared to wild type. Parameters have been calculated according to the scheme. *n*  =  3 wild-type and 3 *Mdr1* mutant mice (each dot represents an oocyte; total wild-type oocytes = 29; total mutant oocytes = 40). (D) Representation of wild-type and *Mdr1* mutant MII oocyte chromosome spreads and their relative aneuploidy frequency. The numbers of chromosomes are indicated; scale bar = 10 μm. *n*  =  13 wild-type oocytes and *n*  = 20 mutant oocytes from three wild-type and three *Mdr1* mutant mice. Unpaired *t*-test; *P < 0.05.

Having observed that MDR-1 mutation causes a delay in GVBD and an abnormal meiotic division, we investigated whether MDR-1 is required for the assembly of the meiotic spindle and proper alignment of the chromosomes at the metaphase plate. To address this question, wild-type and *Mdr1* mutant oocytes were stained for spindle and chromosome organization. As shown in Fig. 2A and B, we found a higher frequency of oocytes having misaligned chromosomes in *Mdr1* mutant oocytes (34 ± 4.5 vs 15 ± 2, *P < 0.05, *n*  =  ~40 oocytes). We also observed that mutant oocytes presented a malformed spindle having an elongated shape (Fig. 2C) (39 µm vs 46 µm ***P < 0.001, wild type and mutant, respectively). In contrast, wild-type metaphase oocytes (*n*  =  ~30) displayed a typical bipolar barrel-shaped spindle and well-aligned chromosomes on the equatorial plate (width at the poles: 10.4 µm vs 10.6 µm and width at the metaphase plate: 24 µm vs 26.5 µm, **P < 0.01, wild type and mutant, respectively). Furthermore, to check whether these meiotic defects in *Mdr1* mutant oocytes would lead to oocyte aneuploidy, we analyzed the number of chromosomes of MII oocytes by chromosome spread (Fig. 2D). Indeed, a higher percentage of *Mdr1* mutant oocytes presented aneuploidy compared to the wild-type ones (14% vs44% in wild-type and mutant oocytes, respectively, *P < 0.05).

**Figure 3:**
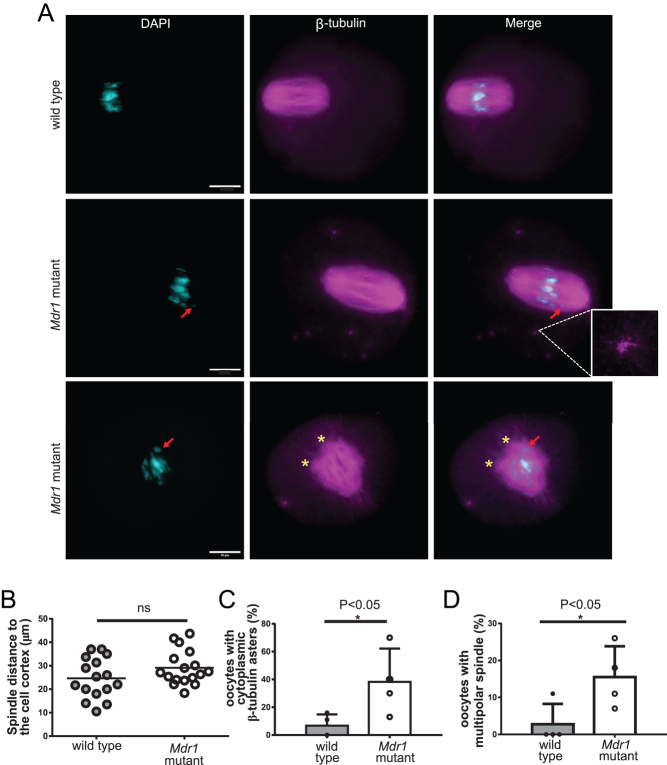
*Mdr1* mutant oocytes exhibit impairment in spindle migration to the cortex, presence of multiple β-tubulin asters in the cytoplasm and a multipolar spindle. (A) Whole-mounted oocytes were stained with anti-tubulin antibody (magenta) for the spindle and chromosomes counterstained with DAPI (cyan). The red arrow indicates misaligned chromosomes, while the yellow asterisks indicate the multipolarity of the spindle and the enlargement shows a β-tubulin aster. Scale bar= 20 µm. *n*  =  3 wild-type and 3 *Mdr1* mutant mice. (B) A higher number of *Mdr1* mutant oocytes have a centered positioned metaphase spindle compared to the wild-type ones (28 µm vs 24 µm, distance to the cortex in mutant and wild-type oocytes, respectively; unpaired *t*-test, ns). Each dot represents an oocyte. (C) Bar chart representing a higher percentage of *Mdr1* mutant oocytes with excessive β-tubulin polymerization and aster formation (47% vs 9% respectively; unpaired *t*-test, *P < 0.05). (D) Bar chart representing a higher percentage of *Mdr1* mutant oocytes with multipolar spindle (22% vs 0% respectively; unpaired *t*-test, *P < 0.05). *n*  =  4 wild-type and 4 *Mdr1* mutant mice.

To assess if the symmetrical division observed in *Mdr1* mutant oocytes (Fig. 1E and F) is due to a lack of the spindle migration from the center of the cell to the cortex, we analyzed wild-type and mutant oocyte spindles and measured the distance between the spindle pole to the cell cortex. We observed that the average distance of the spindle to the cortex was higher in mutant oocytes compared to the wild-type ones (Fig. 3B) (29 ± 2 µm vs25 ± 2 µm, respectively). Moreover, in mutant oocytes, microtubule fibers were excessively polymerized, leading to the formation of a multitudinous number of β-tubulin asters and a multipolar spindle in the cytoplasm (Fig. 3A, C and D) (percentage of oocytes having multiple β-tubulin asters: 7 ± 4% vs 38 ± 12%, *P < 0.05 and percentage of multipolar spindle in wild-type and mutant oocytes: 2.7 ± 2.7% vs 15.5 ± 4%, *P < 0.05).

#### Oocyte Ca^2+^ homeostasis is abnormal in oocytes with aberrant MDR-1

**Figure 4:**
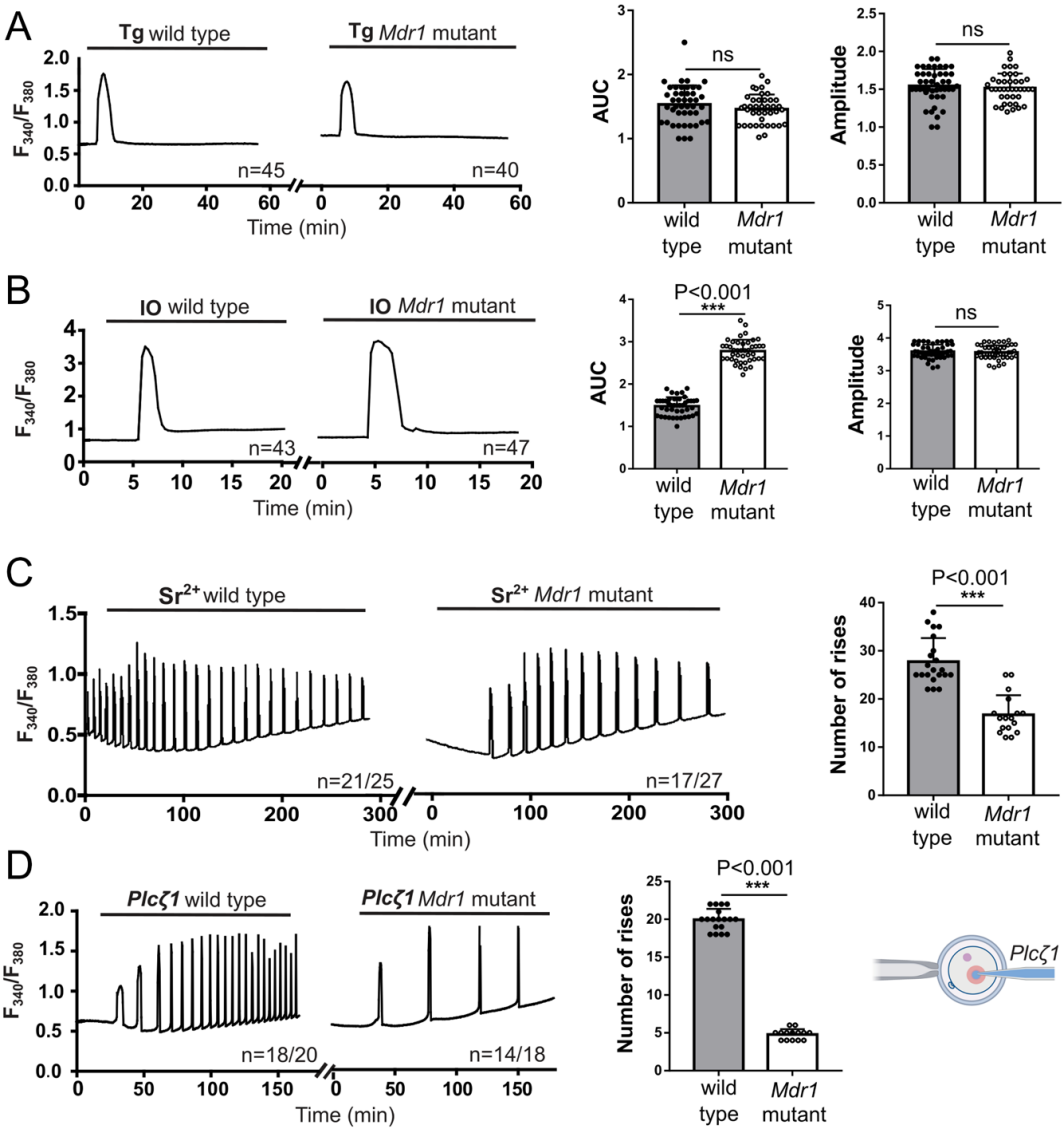
Metaphase II (MII) *Mdr1* mutant and wild-type oocytes present different Ca^2+^ oscillations upon strontium, ionomycin (IO), thapsigargin (TG) and PLCZ1 treatment. (A) When 10 µM TG was used, no differences were observed between wild-type and *Mdr1* mutant cells. (B) After oocyte treatment with 2.5 µM IO, wild-type oocytes were characterized by a quick Ca^2+^ depletion; on the other hand, *Mdr1* mutant oocytes presented a broader Ca^2+^ transient (*t*-test, ***P < 0.001). No difference was observed in the peak amplitude. (C) Treatment with 10 mM strontium chloride (SrCl^2+^) induces Ca^2+^ oscillations with a lower number of rises in the mutant oocytes and a delay in the oscillations (*t*-test, ***P < 0.001). (D) In mutant oocytes, 0.01 µg/µL *PLCZ* mRNA induced Ca^2+^ oscillations characterized by a lower frequency in comparison to the wild type (*t*-test, ***P < 0.001). A schematic representation of the procedure is reported. *n*  =  3 wild-type and 3 *Mdr1* mutant mice. The number of oocytes is reported under each graph per individual treatment.

Next, we assessed different features of Ca^2+^ homeostasis in wild-type and mutant superovulated MII oocytes, including the ability to mount oscillations. We first examined the parameters of the internal Ca^2+^ stores using the sarco/endoplasmic reticulum Ca2+-ATPase (SERCA) pump inhibitor TG. Oocytes were treated with 10 µM TG, and the induced Ca^2+^ release reflects the Ca^2+^ content of the endoplasmic reticulum (ER). Total Ca^2+^ release from the ER is not altered in the absence of functional MDR-1 protein, as the amplitude and duration of the rises induced by TG were similar (Fig. 4A). To evaluate the total Ca^2+^ internal content in oocytes, we employed the Ca^2+^ ionophore, IO. Exposure to IO in the absence of external Ca^2+^ causes an intracellular Ca^2+^ increase, reflecting the content of other organelles besides the ER. *Mdr1* mutant oocytes had a larger Ca^2+^ rise, which included a significant greater area under the curve (AUC) compared to the wild type (Fig. 4B) (wild type = 1.48 AUC vs mutant = 2.77 AUC, ***P < 0.001), which reflects internal Ca^2+^ accumulation, most likely in the mitochondria. This Ca^2+^ accumulation may impair the function of this organelle. We next examined the ability of these oocytes to mount oscillations. We used two approaches: first, we exposed oocytes to 10 mM strontium chloride (Sr^2+^). Sr^2+^ efficiently initiates oscillations in oocytes by activating the inositol 1,4,5-trisphosphate receptor (Tomashov-Matar *et al.* 2005). Fewer *Mdr1* mutant oocyte-initiated oscillations, 63% vs 84% of wild-type oocytes, and the oscillations were considerably delayed in mutant oocytes. Last, the frequency and amplitude of the oscillations were reduced in *Mdr1* mutant oocytes compared to the wild type (Fig. 4C) (*n*  = 27 vs *n*  = 17 number of rises in wild-type and mutant oocytes, respectively, ***P < 0.001). Second, to mimic the fertilization-initiated oscillations, we microinjected oocytes with 0.01 µg/µL phospholipase C-zeta (*PLCζ1*) mRNA, a sperm-specific phospholipase C. Consistent with previous results, mutant oocytes displayed abnormal and reduced frequency of oscillations (Fig. 4D, *n*  = 20 vs *n*  = 5 number of rises in wild-type and mutant oocytes, respectively, ***P < 0.001). This result indicates the inability of mutant oocytes to initiate and sustain Ca^2+^ oscillations, possibly reflective of abnormal intra-organelle Ca^2+^ homeostasis, which may compromise the levels of ATP in the mutant oocytes.

#### The bioenergetics (ATP) of the oocyte is potentially perturbed with MDR-1 dysfunction

The delay observed in GVBD, altered Ca^2+^ homeostasis and our previous finding that NADP+ is significantly reduced in *Mdr1* mutant ovaries (Clark *et al.* 2019) (and that NADP+ production is NAD+ and ATP dependent) (Hashida & Kawai-Yamada 2019) encouraged us to test ATP levels in both *Mdr1* mutant and wild-type oocytes. Lack of MDR-1 function resulted in a trend of ~20% reduction in ATP levels compared to wild-type oocytes; however, this was not significant (Supplementary Fig. 1) (90 ± 6.4 vs 70±7.4 pmol/oocyte, *P* = 0.059), indicating some impairment of metabolic function.

#### Exomic and mitochondrial DNA have increased SNPs in Mdr1 mutant oocytes

Oxidative phosphorylation leads to the generation of reactive oxygen species (ROS) and potentially ovotoxic by-products. Recently, we have shown that MDR-1 may facilitate the export of these by-products like ROS at the germinal vesicle (GV) oocyte stage (Clark *et al.* 2019). mtDNA is susceptible to damage by ROS because it lacks genes involved in DNA repair (García-Lepe & Bermúdez-Cruz 2019), robust repair enzymes and introns (Cline 2012, Napoli *et al.* 2013, Singh *et al.* 2015). It has been established that the mutated mtDNA is associated with abnormal metabolism (Lowell & Shulman 2005, Schilf *et al.* 2021). To assess the effects of MDR-1 mutation and accumulation of ROS on genomic and mtDNA stability, we performed whole exomic and mtDNA sequencing on mutant and wild-type aged oocytes. We observed 7000 more single-nucleotide polymorphisms (SNPs) in the exomic DNA of the mutant oocytes compared to the wild-type ones (Supplementary Fig. 2A). Sequencing of mtDNA from MDR-1 mutant oocytes revealed double the number of SNPs than wild-type age-matched control oocytes (Supplementary Fig. 2B). All the mutations were in the first step of the electron transport complex I, the NADH ubiquinone oxidoreductase gene subunits; the most abundant SNP is a T to C transition in the *Nd5* gene with an allelic frequency of 30% (Supplementary Fig. 2C).

### Premature ovarian aging/diminished ovarian reserve

#### Sirtuin levels are significantly reduced in aged mutant ovaries with MDR-1 loss of function

**Figure 5:**
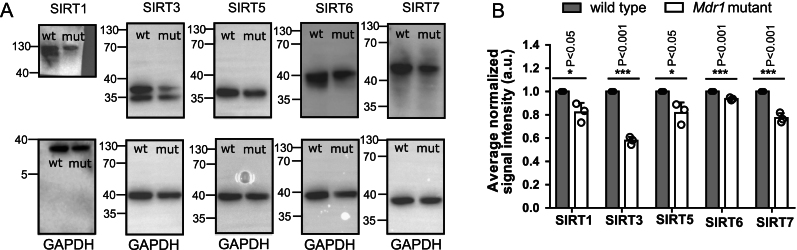
Western blot analysis on sirtuins in 1-year-old ovaries. Whole ovary Western blot analysis shows a reduction in sirtuin protein expression in ovaries isolated from 1-year-old *Mdr1* mutant mice compared to wild type. *n*  =  3 wild-type and 3 *Mdr1* mutant mice. The whole ovary SIRTs band intensity has been calculated using ImageJ software; the ratio of sirtuin/GAPDH expression has been normalized. Unpaired *t*-test was performed, and the respective *P*-values are indicated on the graph. mut, *Mdr1* mutant; wt, wild type.

Our previous finding on the accumulation of ROS and metabolism defects in *Mdr1* mutant oocytes (Clark *et al.* 2019) coupled with the functional relevance of sirtuins in mitochondrial bioenergetics and oxidative stress brought us to the hypothesis that they might be an important downstream mediator of MDR-1 in controlling the oocyte quality of aged oocytes. Moreover, all members of the sirtuin family have a role in maintaining the cell’s metabolic homeostasis, stress response and aging (Ahn *et al.* 2008, Nakagawa *et al.* 2009, Sidorova-Darmos *et al.* 2014, Singh *et al.* 2018), and their alterations lead to metabolic defects, reduced ATP production and accelerated senescence (German & Haigis 2015, Parihar *et al.* 2015, Zhang *et al.* 2020). To test this scenario, we evaluated the expression levels of the two mitochondrial sirtuins SIRT3 and SIRT5 and three nuclear ones SIRT1, 6 and 7 among wild-type and mutant ovaries isolated from aged mice and compared to each other. We found that SIRT1, 3, 5, 6 and 7 protein levels were significantly reduced in MDR-1 mutant aged ovaries compared to age-matched wild-type ovaries (Fig. 5) (SIRT1 *P < 0.05, SIRT3 ***P < 0.001, SIRT5 *P < 0.05, SIRT6 ***P < 0.001 and SIRT7 ***P < 0.001).

#### Loss of MDR-1 function causes a reduction in ovulated oocytes and a lower fertility index

**Figure 6:**
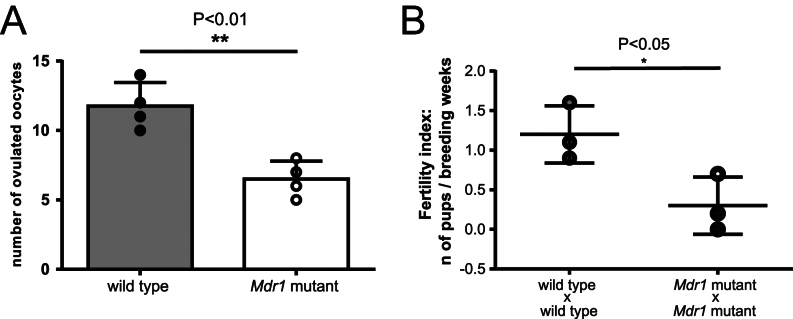
MDR-1 aged mutant females show an early onset of DOR and subfertility. (A) Number of ovulated oocytes (unpaired *t*-test, **P < 0.01), *n*  =  4 wild-type and 4 *Mdr1* mutant mice. (B) Dot plot showing the fertility index of wild-type and mutant breeding pairs. Each dot represents a breeding pair. There is a significant difference between the fertility index of wild type and mutant breading pairs (unpaired *t*-test, *P < 0.05).

Given the lower sirtuin levels in aged mutants and our previous data that young *Mdr1* mutants ovulated double the number of oocytes compared to wild types (Clark *et al.* 2019), we next endeavored to observe the consequences on the ovarian reserve as the mouse aged. We proceeded with the quantification of ovulated oocytes and with the analysis of the fertility index in aged mice. Older mutant animals ovulated fewer oocytes (Fig. 6A) (*n*  =  12 ± 0.85 wild type vs *n*  = 6.5 ± 0.65 mutant, **P < 0.01) and were characterized by a lower fertility index (Fig. 6B) (*n*  =  1.2 ± 0.21 wild type vs *n*  = 0.3 ± 0.21 mutant, *P < 0.05) compared to the wild types. To investigate the presence of senescence in follicles, we stained different sections of wild-type and mutant ovaries with β-galactosidase (X-gal), a marker for senescent cells. We did not find a significant increase in the number of X-gal-positive granulosa cells in the mutant ovary compared to the wild type (Supplementary Fig. 3A and B).

## Discussion

Poor oocyte quality and ovarian aging can clearly compromise the fertile life span of a woman (Broekmans *et al.* 2009, Liu *et al.* 2011, Lord & Aitken 2013). Although significant advances in assisted reproductive technology (ART) have been made, such as early elective egg freezing, oocyte and/or embryo donation, these do not treat the actual underlying cause of premature reproductive aging in individuals. At present, there is no screening or effective treatment because there is a dearth of research and resources expended to understand oocyte biology (Sutherland & McLaughlin 2021). It should also be noted that many more women may not have access to such specialized and costly medical care required to have ovulation induction and subsequent oocyte retrieval (Summers *et al.* 2014, Chiware *et al.* 2021). Furthermore, poor oocyte quality and premature ovarian aging herald a multitude of overall medical problems such as early-onset heart disease (Quinn & Cedars 2018) and low bone density (Cardozo *et al.* 2011, Li & Wang 2018). Therefore, the ovary should be regarded as the ‘canary in the coal mine’ or rather a harbinger of premature somatic aging and reduced longevity. With the growing rate of DOR diagnosed in women, it is imperative that there is a basic understanding of the mechanisms of poor oocyte quality. The aged mutant females present an interesting animal model for DOR as aged animals ovulate fewer oocytes after superovulation compared to age-matched wild-type controls (Fig. 6). However, we previously found that young mutants naturally superovulate and despite a higher yield of oocytes had the same number of pups, suggesting early-onset poor oocyte quality (Clark *et al.* 2019). Our previous work has established that MDR-1 was expressed in the oocyte mitochondrial membrane and is essential for oocyte mitochondrial physiology (Clark *et al.* 2019). In this manuscript, we studied how loss of MDR-1 affects the essential job of the oocyte, which is to resume meiosis, prepare for fertilization and eventually sustain the development of the embryo until implantation (Coticchio *et al.* 2004). These processes require significant energy expenditure facilitated by mitochondria which is the absolute indicator of oocyte quality (Stojkovic *et al.* 2001, Nagano *et al.* 2006). However, the future of reproductive medicine relies on a non-invasive assay to determine oocyte quality and assess energy production via mitochondrial physiology in real time. Our results suggest that MDR-1 functionality could be a candidate biomarker of oocyte quality and ovarian aging.

We demonstrate that MDR-1 is implicated in the control of the quality of aged oocytes likely through modulating sirtuin protein expression or activity. Sirtuins 1–7 are an evolutionarily conserved family of proteins involved in metabolic regulation, protection from ROS, DNA repair and senescence via NAD+ deacetylase and ADP-ribosyltransferase (Nemoto *et al.* 2005, Sosnowska *et al.* 2017, Singh *et al.* 2018). SIRT1 is nuclear and then localizes to the meiotic spindle (Chang & Guarente 2014, Nevoral *et al.* 2019). SIRT6 and SIRT7 are present only in the nucleus and are involved in many processes, including DNA repair, ribosome biogenesis and modulation of inflammation (Barber *et al.* 2012, Kugel & Mostoslavsky 2014)*.* Finally*,* SIRT3 and SIRT5 are present in the mitochondria and regulate all complexes of the electron transport chain (ETC), the urea cycle, purine metabolism and fatty acid oxidation (Nakagawa *et al.* 2009, Parihar *et al.* 2015, Sosnowska *et al.* 2017, Shen *et al.* 2020). Our data show that SIRT1, SIRT3, SIRT5, SIRT6 and SIRT7 are all reduced in the 9- to 12- month-old ovarian lysates of mutants (Fig. 5). SIRT1’s reduction in the ovary of *Mdr1* mutants and involvement in meiosis enabled us to hypothesize that meiosis in the setting of MDR-1 loss would be abnormal. In fact, we observed that MDR-1 dysfunction significantly delays GVBD (Fig. 1) even if it does not affect the overall meiotic resumption. In the literature, a similar pattern has already been described and linked with poor oocyte quality (Ezoe *et al.* 2015, Kumar *et al.* 2018). In particular, Kumar* et al*. in 2018 observed that a delayed GVBD coincides with oocytes with a lower developmental rate. They suggested that this delay could be explained by the fact that lower quality oocytes remain in their preparatory phase longer to gain capability and accumulate all transcripts needed to reach the GVBD. Our observation of aberrant calcium homeostasis in mutant oocytes (Fig. 4) and the emerging evidence that mitochondrial calcium levels are involved in resumption of meiosis (Tiwari *et al.* 2017) lends further credence to how GVBD delay and poor oocyte quality are interrelated. A further explanation might be that GVBD is a high-energy process (Dalton *et al.* 2014) and compromised mitochondria in mutant oocytes (Clark *et al.* 2019) potentially have abnormal ATP production, consequently delaying the resumption of meiosis. Indeed, we observed a trend for pooled mutant GV oocytes to have reduced ATP production, although this was not significant (Supplementary Fig. 1). The next highly energetic step in oocyte maturation is the meiotic spindle formation and its migration to the cortex of the oocyte for the appropriate expulsion of PBI (Zhang *et al.* 2006). The aforementioned events are compromised in MDR-1 mutant oocytes which present an abnormal, more elongated shape rather than the typical murine barrel-shaped meiotic spindle (Fig. 2) and exhibit impaired spindle migration to the cortex (Fig. 3). Moreover, the MDR-1 mutant spindle harbors misaligned chromosomes at its metaphase plate, indicating the weakness of the spindle filaments to attach the kinetochores which can lead to aneuploid eggs as we observed in mutant oocytes (Fig. 2). DOR has clinically been found to correlate to a reduced number of euploid embryos regardless of the maternal age (Jaswa *et al.* 2021). Our findings also demonstrate that *Mdr1* mutants extrude symmetric polar bodies (Fig. 1), which is likely due to perturbed actin dynamics in poor-quality oocytes (Ebner *et al.* 2000, Navarro *et al.* 2009). All these results together potentially suggest that MDR-1 is involved in normal resumption and demarche of meiosis.

It is still unclear how MDR-1 functions exactly in the mitochondrial membrane; however our previous work showed that GV oocytes of *Mdr1* mutants had elevated levels of ROS (Clark *et al.* 2019). It has been recently reported that early-stage oocytes have low complex I assembly and activity and therefore maintain low ROS levels (Rodríguez-Nuevo *et al.* 2022). It is well documented that mtDNA is susceptible to damage, such as ROS, for many reasons including lack of histones and a self-DNA repair mechanism (Cline 2012, Napoli *et al.* 2013, Singh *et al.* 2015). While the mitochondrial genome only codes for 37 genes, 13 of which are proteins, these proteins are essential to the ETC (Taanman 1999). Therefore, mtDNA mutation is clearly associated with abnormal bioenergetics of a cell (Zhang *et al.* 2017, Yan *et al.* 2019). An increased number of mtDNA mutations observed in MDR-1 mutant oocytes (Supplementary Fig. 2), especially in the mitochondrial complex I genes, indicates the importance of MDR-1 in regulating mitochondrial homeostasis and its protection from DNA damage. It has been shown that SIRT3 loss results in a reduction in basal ATP levels in multiple organs, hyperacetylation of complex I components and reduction in complex I activity (Ahn *et al.* 2008). SIRT6 is reported to have a crucial role in maintaining genomic stability and repairing DNA damages by different mechanisms (Kugel & Mostoslavsky 2014) and SIRT7 has a positive influence on mitochondrial homeostasis (Blank & Grummt 2017). Together, the alteration of these sirtuin protein levels in *Mdr1* mutant oocytes, meiotic abnormalities and an increase in the mtDNA damage suggest a possible connection between MDR-1 functionality and oocyte quality. Our future direction will be to elucidate the mechanism of MDR-1 in mitochondrial physiology. This is a fundamental step to one day improving our understanding of DOR and getting closer to enhanced ART options and improved reproductive outcomes.

## Supplementary Material

Supplementary Video 1. High frequency of symmetrical division in Mdr1 mutant oocytes. A. Polar body extrusion (PBE) in a wild type oocyte. B. PBE in an MDR-1 mutant oocyte. scale bar= 20 μm. n= 3 wild type and 3 Mdr1 mutant mice.

Supplementary Video 2

Supplementary Figure 1. Intracellular ATP contents in 2/4-month-old GV oocytes.

Supplementary Figure 2. Exomic and mitochondrial DNA sequencing. A.

Supplementary Figure 3. β-Galactosidase (X-Gal) staining of wild type and mutant ovarian sections A,B.

Supplementary Figure 4. Immunoblot analysis on Anti-P Glycoprotein in 4-5 month old pooled GV oocytes.

## Declaration of interest

L M B is the Chief Medical Officer for Clue by Biowink, GmbH a software as medical device company. The other authors have no conflicts of interest.

## Funding

This work was funded by the Global Consortium for Reproductive Longevity and Equality.

## Data availability

The data that support the findings of this study are openly available in Figshare at https://doi.org/10.6084/m9.figshare.19705414. All other relevant data supporting the key findings of this study are available within the article and its Supplementary information files or from the corresponding author upon reasonable request.

## Author contribution statement

D N, D B, R F and L M B contributed to writing the manuscript; D N, D B, N G and N T contributed to generating the data included in this manuscript; all authors contributed to the design and/or supervision of the work.
