# Supplementary figures and images for: Multidrug resistance transporter-1 dysfunction perturbs meiosis and Ca2+ homeostasis in oocytes
Source: Reproduction. 2022 Oct 10;165(1):79–91. doi: 10.1530/REP-22-0192 (PMC9782432; doi:10.1530/REP-22-0192)

# oocyte ATP quantification

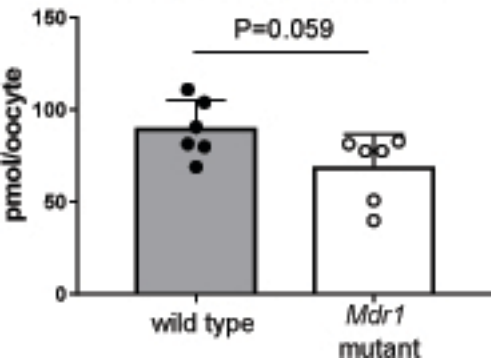

Supplement: Supplementary Figure 1. Intracellular ATP contents in 2/4-month-old GV oocytes. [file supplementary_figure_1.pdf]

**A.**

Exomic DNA: SNPs number

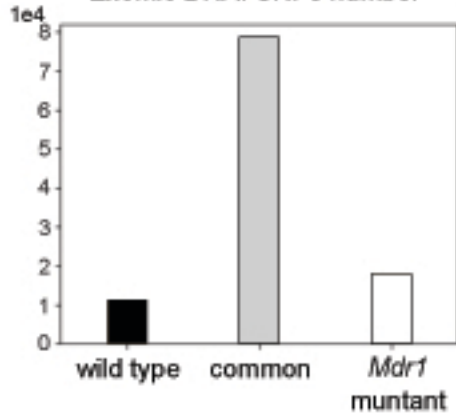**B.**

mtDNA: SNPs number

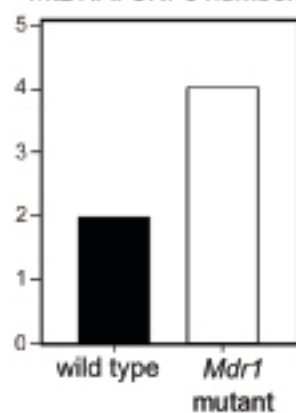**C.**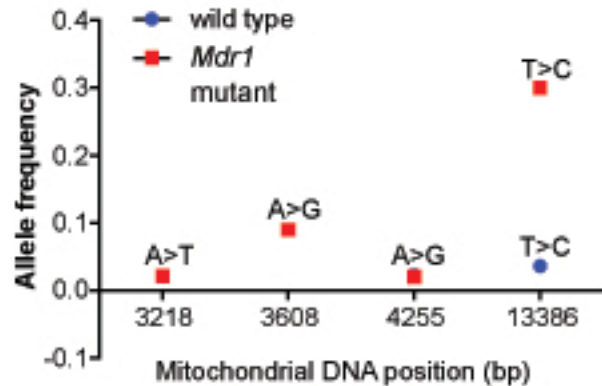

Supplement: Supplementary Figure 2. Exomic and mitochondrial DNA sequencing. A. [file supplementary_figure_2.pdf]

A.

wild type

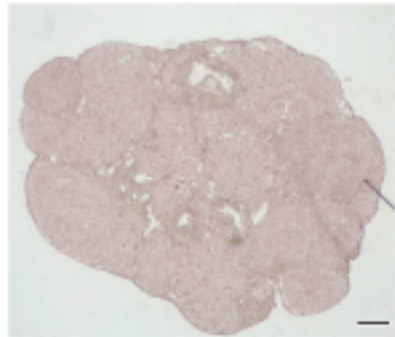*Mdr1* mutant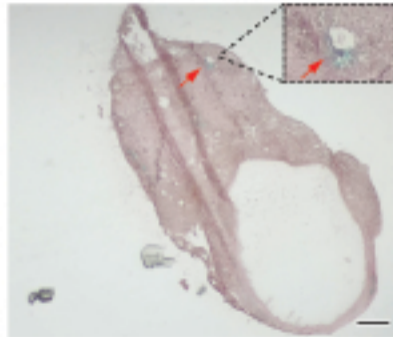

B.

X-Gal positive follicles

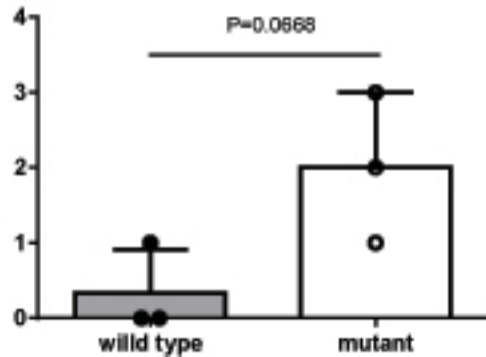

Supplement: Supplementary Figure 3. β-Galactosidase (X-Gal) staining of wild type and mutant ovarian sections A,B. [file supplementary_figure_3.pdf]

Wild type  
MDR1 mutant

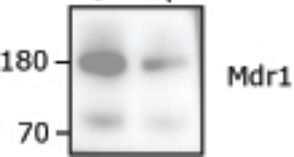

Supplement: Supplementary Figure 4. Immunoblot analysis on Anti-P Glycoprotein in 4-5 month old pooled GV oocytes. [file supplementary_figure_4.pdf]
